# Correction to Competing Interests

**DOI:** 10.1186/s42466-023-00242-y

**Published:** 2023-04-25

**Authors:** Neurological Research and Practice

**Affiliations:** London, UK

## Correction: Neurological Research and Practice (2023) 5:3. 10.1186/s42466-022-00229-1, Neurological Research and Practice (2023) 5:6; 10.1186/s42466-023-00232-0, Neurological Research and Practice (2023) 5:8. 10.1186/s42466-023-00234-y

Following publication of the original article [1–3], it is noticed that the editors as authors should be mentioned in the Competing Interests section.

The following sentences should be added to the Competing Interests section in article [1]:

TR and PR are members of the editorial board of Neurological Research and Practice.

The following sentences should be added to the Competing Interests section in article [2]:

AG is a member of the editorial board of Neurological Research and Practice.

The following sentences should be added to the Competing Interests section in article [3]:

US is a member of the editorial board of Neurological Research and Practice.

The original articles [1–3] have been updated.
